# Bioengineering extracellular vesicles: smart nanomaterials for bone regeneration

**DOI:** 10.1186/s12951-023-01895-2

**Published:** 2023-04-27

**Authors:** Kenny Man, Neil M. Eisenstein, David A. Hoey, Sophie C. Cox

**Affiliations:** 1 School of Chemical Engineering, University of Birmingham, Birmingham, B15 2TT UK; 2 Research and Clinical Innovation, Royal Centre for Defence Medicine, ICT Centre, Vincent Drive, Birmingham, B15 2SQ UK; 3 Institute of Translational Medicine, University of Birmingham, Heritage Building, Mindelsohn Way, Birmingham, B15 2TH UK; 4 Trinity Centre for Biomedical Engineering, Trinity Biomedical Sciences Institute, Trinity College, Dublin, D02 R590 Ireland; 5 Dept. of Mechanical, Manufacturing, and Biomedical Engineering, School of Engineering, Trinity College, Dublin 2, D02 DK07 Dublin, Ireland; 6 Advanced Materials and Bioengineering Research Centre, Trinity College Dublin & RCSI, Dublin 2, D02 VN51 Dublin, Ireland

**Keywords:** Bone, Extracellular vesicles, Nanomaterials, Bioengineering, Biomaterials, Regenerative medicine

## Abstract

In the past decade, extracellular vesicles (EVs) have emerged as key regulators of bone development, homeostasis and repair. EV-based therapies have the potential to circumnavigate key issues hindering the translation of cell-based therapies including functional tissue engraftment, uncontrolled differentiation and immunogenicity issues. Due to EVs’ innate biocompatibility, low immunogenicity, and high physiochemical stability, these naturally-derived nanoparticles have garnered growing interest as potential acellular nanoscale therapeutics for a variety of diseases. Our increasing knowledge of the roles these cell-derived nanoparticles play, has made them an exciting focus in the development of novel pro-regenerative therapies for bone repair. Although these nano-sized vesicles have shown promise, their clinical translation is hindered due to several challenges in the EV supply chain, ultimately impacting therapeutic efficacy and yield. From the biochemical and biophysical stimulation of parental cells to the transition to scalable manufacture or maximising vesicles therapeutic response in vivo, a multitude of techniques have been employed to improve the clinical efficacy of EVs. This review explores state of the art bioengineering strategies to promote the therapeutic utility of vesicles beyond their native capacity, thus maximising the clinical potential of these pro-regenerative nanoscale therapeutics for bone repair.

**Graphical Abstract:**

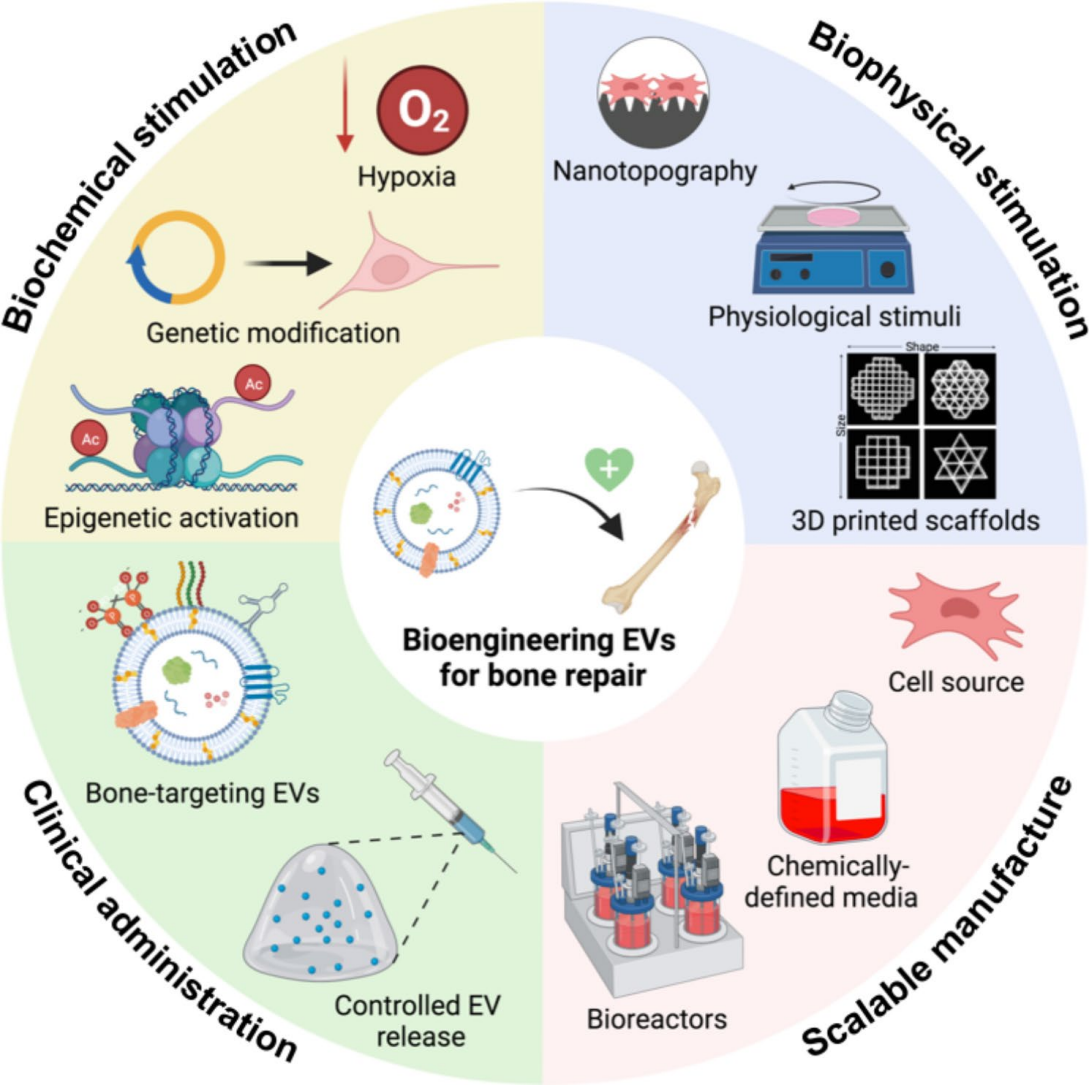

## Introduction

There is an urgent clinical need for effective therapeutic strategies to treat damaged bone caused by traumatic injury, tumour resection or osteoporosis (OP) [1, 2]. Approximately 127 million people in the US are afflicted with musculoskeletal disorders, costing an estimated $213 billion to treat [3]. Moreover, the growing ageing population is expected to further exacerbate the socioeconomic burden of bone-associated disorders. Autograft and allografts are the current gold standard treatments for localised bone loss; however, these therapies are associated with numerous challenges such as prolonged inflammation, donor site morbidity and limited tissue availability [4, 5]. Bone graft substitutes in combination with supraphysiologic doses of osteoinductive growth factors such as bone morphogenic protein 2 (BMP2) have been investigated with positive clinical results [6]. However, hyper-concentrated BMP2 concentrations have been shown to result in severe complications such as hematoma, heterotopic ossification, myelopathy and inflammation, which likely require additional surgical intervention [7, 8]. Thus, due to issues with conventional treatments, there is a critical need for novel strategies that can stimulate effective bone tissue regeneration.

There has been an increasing body of evidence demonstrating the trophic effects of cell-secreted bioactive products in regulating cellular communication [9, 10]. Of these factors, extracellular vesicles (EVs) are considered one of the most important secretory products, involved in numerous trophic and immunomodulatory processes [11, 12].

EVs are defined as nanoscale lipid particles that contain a diverse biological cargo of nucleic acids, proteins and bioactive molecules [13–15]. These nanoparticles possess a phospholipid bilayer containing major histocompatibility complex molecules, receptors and tetraspanins (CD9, CD63 and CD81), whilst the lumen contains enzymes, proteins, nucleic acids and other signalling molecules [14, 16]. EVs are generally classified into three subtypes based on their biogenesis, composition and size. Exosomes (30–150 nm) are formed from the endosomal route and are released when multivesicular bodies fuse with the plasma membrane [17]. Microvesicles (50–1000 nm) are created from the outward blebbing of the cell membrane [18]. Apoptotic bodies (500–2000 nm) are highly heterogenous and are formed during apoptosis from the plasma membrane [19]. The intercellular communication via the delivery of these EV-associated bioactive factors is critical in mediating biological functions between cells [20, 21]. The rapid recent developments in the EV field [22], signify their potential impact on future healthcare technologies. Furthermore, the diverse biological cargo of EVs may enhance therapeutic benefits when compared to other nano-sized delivery systems such as synthetically-derived nanoparticles and liposomes. For instance, EVs exhibit innate biocompatibility, low immunogenicity, and high physiochemical stability, key issues hindering the clinical utility of other synthetic or bioinspired mimetics nanomaterials [13, 18, 23]. Thus, these naturally-derived nanoparticles have garnered growing interest as potential nanoscale therapeutics for a variety of diseases [24, 25].

Within the bone context, several studies have reported the intrinsic role of EVs in regulating bone homeostasis by mediating intercellular communication [26, 27]. Moreover, it is thought that these cell-derived nanoparticles are fundamentally involved in bone development, as extracellular matrix bound vesicles are critical for endochondral ossification [28, 29]. Increasing evidence has demonstrated the comparable regenerative capacity of EV-based therapies when compared to cell-based treatments for the repair of large bone defects in vivo [30, 31]. Thus, harnessing EVs for regenerative medicine is an attractive acellular, but biological approach to recapitulate the complex process of bone tissue repair. Excitingly, these EV-based therapeutics have the potential to circumnavigate key issues hindering the translation of cell-based therapies, such as their inherent heterogeneity, functional tissue engraftment, uncontrolled differentiation, immune rejection and physiochemical instability [32–34]. Moreover, EVs are advantageous when compared to similarly sized synthetic drug delivery systems as they exhibit reduced clearance rates, enhanced circulation times and reduced risked of systemic toxicity [18].

Despite the growing promise of vesicles as acellular tools to stimulate bone repair, their clinical adoption has been hindered due to issues including the selection of an appropriate cell source, EVs inherent therapeutic efficacy, scalable manufacture and their clinical administration [13]. In this review, we will discuss the current status of EVs for bone regeneration, highlight novel bioengineering strategies to promote their clinical efficacy and identify key issues in the field hindering their clinical translation.

## Extracellular vesicles in the bone microenvironment

**Fig. 1:**
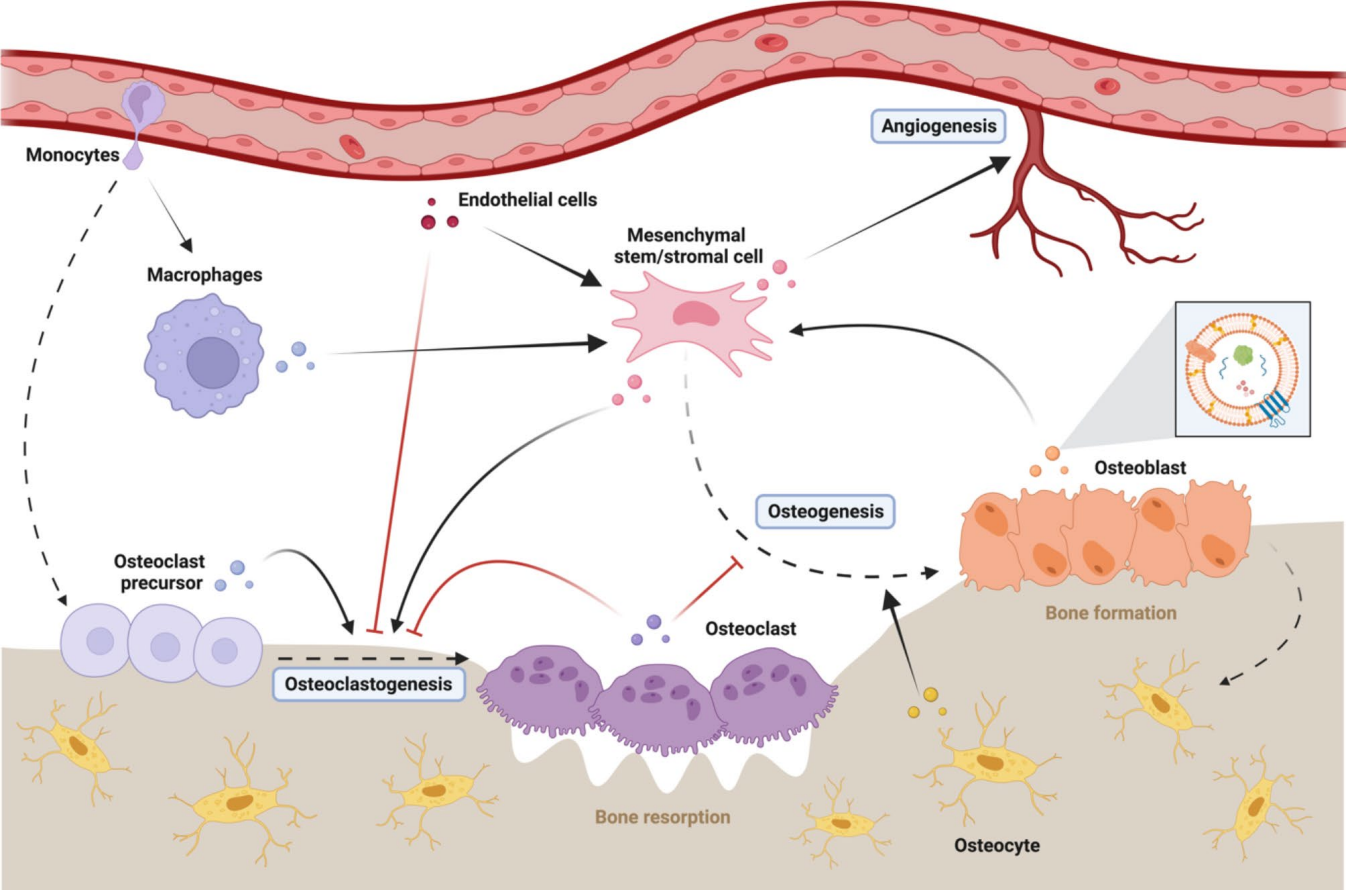
Schematic representation of the diverse role of cell secreted EVs in the bone microenvironment and regulation of bone homeostasis. Promotion (black arrows), Inhibition (red lines) and differentiation (black dash arrow) pathways

The bone microenvironment consists of a diverse subset of cell types including mesenchymal stem/stromal cells (MSCs), osteoblast, osteoclast, osteocytes, macrophages, endothelial cells (ECs), and many more within the marrow, which synergistically regulate bone homeostasis [35, 36]. Due to the complexity of the bone microenvironment, there have been extensive investigations aiming to elucidate the role of cell-specific EVs on the bone remodelling process (Fig. 1).

### MSC-derived EVs

MSCs are an attractive cell population used in bone augmentation due to their ease of procurement from numerous tissues and their ability to differentiate into multiple lineages [37, 38]. Within the bone microenvironment, MSCs are the precursors to osteoblasts, which are the cell type responsible for bone formation (Fig. 1) [39]. Although, the therapeutic applicability of MSC-based therapies have been reported [40], their clinical translation is hindered by several challenges including their inherent heterogeneity, low transplanted cell viability, immune rejection, uncontrolled differentiation and teratoma formation [41–43]. The beneficial effects exerted by MSCs are now thought to be due to the paracrine factors they secrete which have numerous trophic and immunomodulatory effects [44–46]. Thus, cell-free strategies harnessing the bioactive products within MSC-EVs represents a promising approach to stimulate bone regeneration, overcoming the limitations regarding the translation of MSC-based therapies. As such, there has been growing evidence demonstrating the therapeutic efficacy of MSC-derived EVs for bone repair [47, 48]. For instance, Jiang et al. described the role of miR-25 enriched in bone marrow derived-MSCs (bMSC)-EVs in protecting Runx2 from ubiquitination and degradation, ultimately promoting bone fracture healing in mice [49]. Similarly, Zhai et al. demonstrated that EVs-derived from bMSCs were able to promote bone formation by stimulating the PI3K/Akt and MAPK signalling pathways via the upregulation of pro-osteogenic microRNAs and downregulation of anti-osteogenic microRNAs [31].

Vesicles derived from MSCs have also been reported to regulate angiogenesis, a key process involved in bone fracture healing. For instance, Lu et al. demonstrated the role of MSC-EVs in promoting both angiogenesis and osteogenesis in vitro and in vivo [50]. EVs isolated from bMSCs promoted the migration, proliferation and tube formation of human umbilical vein endothelial cells (HUVECs). It was found that angiogenic-related miRNA, miR-29a, was upregulated within bMSC-EVs and was involved in promoting angiogenesis in recipient HUVECs by targeting VASH-1, a negative angiogenic regulator. The delivery of miR-29a-enriched EVs from genetically modified bMSCs, promoted angiogenesis and osteogenesis within a murine model. In a similar approach, Cheng et al. found that Nidogen1, an extracellular matrix protein, were enriched within bMSC-EVs [51]. These vesicles inhibited the formation and assembly of focal adhesions in rat arterial ECs by targeting myosin-10, resulting in promoting the migratory and angiogenic potential of recipient cells. Moreover, when delivered within a hydrogel system, the Nidogen1-enriched EVs enhanced rat femoral defect repair.

MSCs-EVs also exhibit similar immunoregulatory properties to their parental cells during different stages of osteogenesis. For example, Nakao et al. reported that treating human gingiva-derived MSCs with TNF-α, resulted in the production of EVs that enhanced the M2 polarisation of macrophages and inhibited periodontal bone loss [52]. Wei et al. investigated the role of MSC-EVs in regulating the osteoimmune environment [53]. The authors showed that vesicles isolated from osteogenically differentiated bMSCs significantly reduced the expression of proinflammatory genes and M1 phenotypic markers in macrophages. Collectively, these studies highlight the role of MSCs-EVs in regulating the osteoimmune environment.

Extensive research has indicated MSCs derived from different tissue origins exhibit differential capacity to promote bone regeneration [54, 55]. As EVs are essentially fingerprints of their parent cell, there has been increasing studies showing the importance of MSCs tissue origins on EVs pro-regenerative capacity. Wang et al. (2020) conducted proteomics analysis of MSC-derived EVs procured from bone marrow, adipose and umbilical cord tissues [56]. They discovered that vesicles derived from bMSCs were enriched with pro-osteogenic proteins, while EVs from adipose-derived MSCs were loaded with immunoregulatory factors and umbilical cord MSCs-EVs contained proteins involved in mediating tissue repair. Another key factor impacting the therapeutic efficacy of MSC-derived EVs is the age of the donor. The process of aging is correlated with bone loss and delayed fracture healing [57]. Extensive research has shown that MSCs acquired from younger patients have been reported to elicit enhanced regenerative potential when compared to those procured from older patients [58–60]. Recently, Xu et al. (2020) showed that EVs derived from aged-MSCs exhibited diminished repair capacity to promote healing within a rat femoral fracture model compared to EVs acquired from younger MSCs [61]. Moreover, miRNA analysis confirmed the upregulation of miR-128-3p in aged-EVs, an inhibitor of Smad5, a key osteogenic transcription factor. Taken together, these studies highlight the importance of cell sourcing on the therapeutic efficacy of MSC-EVs for bone repair.

### Osteoblast-derived EVs

Osteoblasts are derived from bone marrow MSCs and are responsible for the synthesis and mineralisation of the bone matrix (Fig. 1) [62, 63]. EVs from mineralising osteoblasts have been shown to promote the osteogenic differentiation of MSCs by activating the Wnt signalling pathway, calcium signalling, and the delivery of pro-osteogenic microRNAs [64]. Several studies have described the differential expression of proteins within osteoblast-derived EVs during osteogenesis. For example, EVs-derived from mineralising osteoblasts were found to be upregulated in factors such as Transforming growth factor beta 3, eukaryotic initiation factor 2, BMP-1, SMAD specific E3 Ubiquitin Protein Ligase 1 (SMURF-1) proteins [65, 66].

Interestingly, Uenaka et al. recently reported vesicles derived from mature osteoblasts inhibited bone formation and enhanced osteoclastogenesis in vivo [67]. EVs were isolated from primary murine osteoblasts over 2-week period in mineralising conditions. These vesicles were enriched with miR-143, which inhibited Runx2 expression and subsequent osteogenesis. Moreover, osteoblast-EVs enhanced the expression of receptor activator of NF-κB ligand (RANKL), thus promoting osteoclastogenesis. This work was supported by Kobayashi-Sun et al. where their findings showed that osteoblast-EVs induced osteoclast differentiation within transgenic zebrafish [68]. Similarly, Deng et al. demonstrated that osteoblast secreted EVs contained RANKL protein, which activated osteoclast formation [69].

Additionally, there is evidence indicating the role of osteoblast-derived EVs in regulating angiogenesis. Tang et al. reported that EVs derived from 21 day osteogenically cultured preosteoblast promoted EC angiogenesis via EV associated MMP activation of the VEGF/Erk1/2 signalling pathway [70]. Collectively, these research findings indicate the diverse roles of osteoblast-derived EVs in regulating bone remodelling.

### Osteoclast-derived EVs

Osteoclasts are multinucleated cells generated from hematopoietic precursors including blood/bone marrow monocytes and macrophages (Fig. 1) [71]. These cells are responsible for the bone resorption activity within the bone microenvironment [72].

Osteoclast-derived EVs have been reported to promote osteoblast differentiation. For example, Chen et al. demonstrated that osteoclast EVs were able to promote the osteogenic differentiation of the mouse preosteoblast KusaO cell line [73]. Contrastingly, Yang et al. showed that EVs derived from osteoclasts were enriched with miR-23a-5p and suppressed osteoblast differentiation via inhibiting Runx2 expression, which regulated Yes-associated protein-1 mediated Metallothionein 1D Pseudogene inhibition [74]. Similarly, Li et al. described the inhibition of osteoblastic bone formation in ovariectomised (OVX) mice via the delivery of miR-214-3p within osteoclast-derived EVs [75]. Furthermore, Sun et al. showed that suppressing osteoclast EVs secretion via Rab27a siRNA, enhanced bone mineral density in OVX mice.

Huynh et al. reported that EVs secreted from precursor cells promoted osteoclastogenesis, whilst mature osteoclast-EVs inhibited osteoclast differentiation [76]. Mature osteoblast-derived EVs were found to be enriched with receptor activator of nuclear factor κB (RANK), thus likely involved in competitively binding to RANKL on the surface of osteoblast, inhibiting RANK-RANKL activation of osteoclastogenesis. Together, these studies demonstrate the complex roles osteoclast-derived EVs have in regulating bone homeostasis.

### Osteocyte-derived EVs

Osteocytes are terminally differentiated cells that are embedded within the mineralised matrix of bone (Fig. 1) [77]. These cells are mechanosensitive, allowing them to respond to specific external stimuli through modifying their trophic factors. Several studies have reported the role of osteocyte secreted EVs in promoting osteogenesis [78, 79]. For example, Lv et al. demonstrated that osteocytes subjected to mechanical strain secreted EVs that stimulated osteogenic differentiation of human periodontal ligament stem cells (hPDLSCs) via the miR-181b-5p/PTEN/AKT signalling pathway [79]. Contrastingly, Qin et al. reported that EVs derived from myostatin-treated osteocytes suppressed osteoblast differentiation through miR-218 mediated inhibition of Runx2 and Wnt signalling [80].

### Endothelial cell-derived EVs

Angiogenesis is an essential process involved in normal fracture healing, with defective blood vessel development associated with poor outcomes [81, 82]. EC-derived EVs have been shown to modulate bone regeneration by stimulating angiogenesis (Fig. 1).

Jia et al. showed during the process of distraction osteogenesis, EVs secreted from ECs were capable of accelerating bone regeneration in rats. Specifically they found that the miR-126 enriched EVs stimulated angiogenesis by targeting the Raf/ERK signalling pathway [30]. Moreover, studies have shown that EC-derived EVs are capable of reversing osteoporotic phenotype. Song et al. reported EVs derived from ECs exhibited enhanced targeting to bone tissue in mice when compared to EVs from MSCs and osteoblasts [83]. Moreover, EC-derived EVs inhibited osteoclast activity in vitro and suppressed the osteoporotic phenotype in OVX mice. MicroRNA sequencing identified the upregulation of miR-155 in EC-EVs, which was found to suppress osteoclast activation. Zhang et al. described the role of EC-derived EVs in preventing osteonecrosis of the femoral head by stimulating osteogenesis via the delivery of miR-27-a in EVs [84]. Together, these studies highlight the diverse role endothelial-derived EVs play in regulating bone homeostasis.

### Macrophage-derived EVs

Macrophages are critical components of the immune response and have been shown to contribute to the regulation of MSC and osteoblast function during bone repair (Fig. 1) [85–87]. At the initial inflammatory stage of bone repair, M1 polarised macrophages secrete a plethora of chemotactic and inflammatory factors, which are involved in the recruitment of multiple cell types such as ECs and MSCs to the injury site [88]. Subsequently, the polarisation of macrophages to the M2 anti-inflammatory phenotype facilitate the osteogenic differentiation of MSCs at the late stage of bone regeneration [89, 90].

Studies have showed that macrophages are able to stimulate MSCs osteogenic differentiation through EV signalling [91]. Kang et al. investigated the role of EVs derived from polarised macrophages on bone regeneration [92]. Rat calvarial defects were treated with vesicles derived from M0, M1 or M2 macrophages. The M0 and M2 EVs promoted bone regeneration, whilst M1 EVs suppressed bone healing. Microarray analysis confirmed the role of polarisation on altering the expression of inhibitory and osteogenic microRNAs within M1 and M2 derived EVs respectively. Similarly, EVs derived from M2 polarised macrophages promoted MSC osteogenesis and inhibited adipogenesis through the miR-690/IRS-1/TAZ signalling axis [93]. Xiong et al. reported miRNA-5106 within EVs-derived from M2 macrophages enhanced MSCs osteogenesis by targeting Salt-Inducible Kinase 2 and 3 [94]. Collectively, these studies demonstrated the role macrophages derived EVs play in regulating the osteoimmune environment.

Taken together, these studies emphasise the diverse roles of EVs within the bone microenvironment in regulating skeletal tissue homeostasis and repair (Table 1).

**Table 1:** Studies investigating the role of EVs within the bone microenvironment on regulating bone regeneration

Bone EV cell source	Bioactive cargo	Study observations	Reference
**bMSCs**	miR-25	Protecting Runx2 from ubiquitination and degradation, enhanced fracture healing	[49]
	miR-146a-5p, miR-503-5p, miR-483-3p, miR-129-5p	PI3K/Akt and MAPK signalling pathways	[31]
	**-**	Decreased pro-inflammatory gene expression and enhanced osteogenesis	[53]
	miR-29a	Promoted angiogenesis in HUVECs by targeting VASH-1	[50]
**Osteoblasts**	miR-3084-3p, miR-680, miR-677-3p, miR-5100	Enhanced osteogenesis by activating Wnt signalling pathway	[64]
	miR-143	Inhibited osteogenesis by targeting Runx2. Enhanced NF-κB expression promoting osteoclastogenesis	[67]
**Osteoclasts**	miR-214-3p	Inhibition of osteoblastic bone formation by inducing osteoclast differentiation via the PTEN/PI3k/AKT pathway	[75]
	miR-23a-5p	Suppressed osteoblast differentiation via inhibiting Runx2	[74]
**Osteocytes**	miR-181b-5p	Osteogenesis of hPDLSCs via the PTEN/AKT signalling pathway	[79]
	Annexin A5, Ywhae, Ywhab	Stimulation of MSC recruitment and osteogenesis	[78]
**Endothelial cells**	miR-126	Accelerated bone regeneration by stimulating angiogenesis by targeting Raf/ERK signalling	[30]
	miR-27-a	Prevented femoral head osteonecrosis by stimulating osteogenesis	[84]
**Macrophages**	miR-155 (M1 EVs) miR-378a (M2 EVs)	M1 EVs inhibited osteogenesis, M2 EVs increased osteogenesis	[92]
	miR-690	M2 EVs promotes osteogenesis and inhibited adipogenesis via IRS-1/TAZ signalling	[93]

## Strategies to improve the clinical application of EVs for bone repair

As cells are highly sensitive to the environment they reside in, researchers have harnessed biochemical and biophysical cues to augment the cells phenotype and ultimately the therapeutic efficacy of the EVs they secreted for bone regeneration.

### Biochemical stimulation

**Fig. 2:**
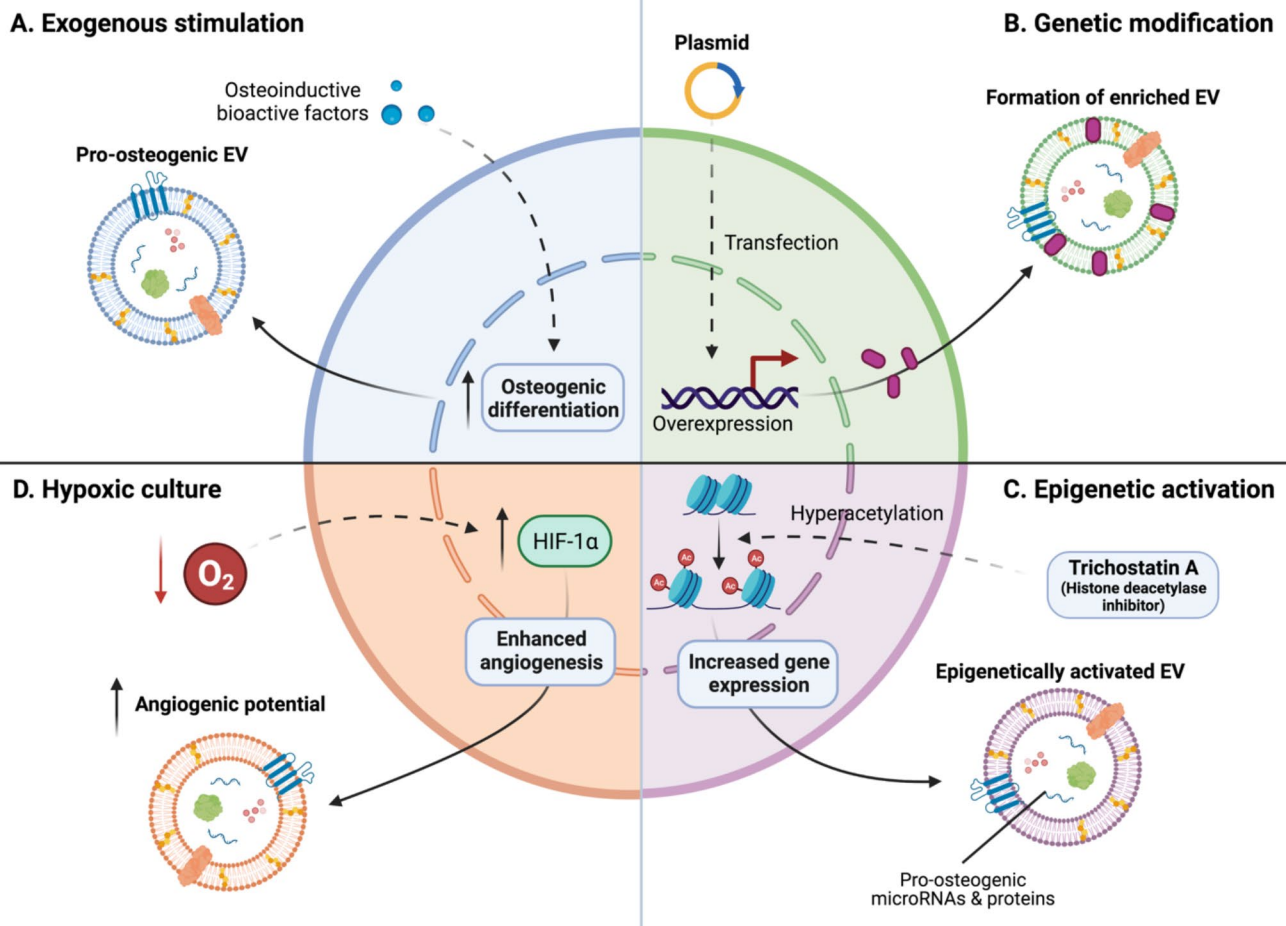
Biochemical stimulation strategies to engineer vesicles with improved pro-regenerative capacity for bone repair. These approaches include (**A**) stimulation through exogenous factors, (**B**) genetic modification, (**C**) epigenetic activation and (**D**) hypoxic conditioning

Whilst there has been growing evidence reporting the regenerative capacity of EVs derived from numerous cell sources, the inherent therapeutic potency of these vesicles is still a factor hindering clinical translation [95]. As EVs are essentially fingerprints of their parental cell, increasing investigations have exploited cell engineering techniques to improve the biological potency of EV-based products [13]. Biochemical stimulation has been demonstrated as an effective strategy to augment the therapeutic function of cells for bone regeneration [96, 97]. In this section, we will describe novel ways in which these approaches have been adopted for engineering EVs, including exogenous stimulation, genetic modification, epigenetic reprogramming and hypoxic culture to improve their potency for bone repair (Fig. 2).

#### Exogenous stimulation

Increasing studies have shown the influence of cell culture parameters in modulating the phenotype of parental cells, ultimately augmenting the therapeutic potency of their EVs. For example, Davies et al. reported the impact of culture media composition on modulating the efficacy of secreted EVs [98]. The authors showed the importance of β-glycerophosphate on the mineralisation of bMSCs treated with osteoblast-derived EVs, thus emphasising the influence of culture media composition on EV-induced mineralisation. The EV parental cell phenotype during isolation is likely dependent on the culture parameters (i.e. media composition, collection frequency/duration). As such, increasing studies have investigated the impact of isolating EVs at different stages of osteogenesis [99]. Wei et al. isolated EVs from osteoblasts at early, mid and mid-late stages of differentiation, and reported that mid-late stage EVs promoted the mineralisation of recipient MSCs in vitro. Moreover, the authors demonstrated that vesicles procured from the mid and mid-late stages of osteoblast differentiation exhibited enhanced bone-targeting potential, via increased accumulation of fluorescently labelled EVs observed in the femurs of mice following tail vein administration. Importantly, the mid-late stage vesicles substantially improved bone mineral density in osteoporotic mice [100]. Researchers have also investigated augmenting EV immunomodulatory function in the context of bone repair [52, 101]. Kang et al. pre-conditioned MSCs with the pro-inflammatory cytokine TNF-α and assessed the osteoimmunomodulatory properties of their EVs [101]. The authors demonstrated that while the native and TNF-α EVs promoted MSC osteogenesis at similar levels in vitro, the TNF-α EVs greatly reduced macrophage pro-inflammatory M1 markers and increased anti-inflammatory M2 markers Importantly, treatment with TNF-α EVs enhanced bone repair within a critically-sized rat calvarial defect. MicroRNA analysis highlighted the enrichment of anti-inflammatory microRNAs in the TNF-α EVs.

Overall, these studies indicate the importance of refining the EV parental cell culture parameters in order to procure vesicles with optimum pro-regenerative capacity (Fig. 2A).

#### Genetic modification

Manipulation of a cell’s genetic makeup is one of the most well-established approaches to augment cell function. Thus, it is not surprising that genetic engineering has been increasingly adopted as a strategy to improve EVs utility for bone repair [13] (Fig. 2). Several studies have demonstrated the promise of this approach in developing pro-osteogenic EVs. For example, Li et al. overexpressed the microRNA miR-101 within MSCs, resulting in enrichment of secreted vesicles [99]. The miR-101-EVs repressed the expression of FBXW7 in recipient MSCs, which in turn enhanced the expression of pro-osteogenic factors hypoxia-inducible factor-1α (HIF-1α) and FOXP3. Similarly, Chen et al. engineered miR-375 enriched EVs via the overexpression within human adipose MSCs [102]. MiR-375 enriched EVs promoted human bMSCs osteogenic differentiation in vitro and enhanced bone regeneration within a rat calvarial defect.

Besides the loading of nucleic acids, the overexpression of pro-regenerative proteins has also been conducted [103–105]. For instance, Li et al. investigated overexpressing the pro-osteogenic transcription factor HIF-1α within bMSCs [104]. The EVs derived from HIF-1α transfected bMSCs were found to promote the osteogenic differentiation of recipient bMSCs and enhanced tube formation of HUVECs. Moreover, the authors observed enhanced angiogenesis and bone regeneration when EVs from HIF-1α transfected bMSCs were administered in rabbit models of glucocorticoid-induced osteonecrosis of the femoral head. Similarly, Huang et al. evaluated the impact of BMP2 overexpression within MSCs on the osteoinductive potency of secreted EVs [105]. The authors found that EVs derived from BMP2 overexpressed MSCs promoted the osteogenic differentiation of recipient MSCs and enhanced the regeneration of rat calvaria defects in vivo when compared to unmodified vesicles. Interestingly, mechanistic studies confirmed the successful overexpression of BMP2 in parental cells, however, the secreted EVs lacked the protein of interest. Further investigations identified microRNAs enriched within these vesicles, which are involved in potentiating BMP2 signalling. Therefore, these studies highlight the possibility to advance osteogenic potency of EVs by overexpressing pro-osteogenic protein. However, the EVs osteoinductive mode of action will be dependent on whether the protein of interest is sufficiently loaded into the vesicle, or other non-specific cargos are enriched in its place.

Although there is growing evidence demonstrating the promise of engineering EVs through genetic modifications, concerns of transduction efficiency remain. The intensive cost and time associated with this approach may also hinders its clinical utility [13]. Moreover, due to safety concerns regarding the process, EVs derived from genetically-modified cells will likely be classed as Advanced Therapy Medical Products (ATMPs), which would require more rigorous testing in regard to its clinical safety when compared to production from non-genetically modified cells [106].

#### Epigenetic reprogramming

In recent years, epigenetic regulation has garnered increasing attention as a critical process in controlling cell fate [107, 108]. Epigenetics involves modifying a cell’s transcriptional activity without altering the underlying DNA sequence [109, 110], therefore potentially bypassing the limitations of genetic modification. Augmenting the acetylation state of the chromatin by modifying histone deacetylase (HDAC) activity and histone acetyltransferase enzymes have been shown to regulate the cells’ transcriptional activity [111]. Chromatin hyperacetylation induced by HDAC inhibition has been reported to stimulate osteogenic differentiation, through the activation of osteoblast-related genes [112–114]. As such, there have been extensive investigations aiming to replicate the epigenetic landscape within cells during osteogenesis by employing epigenetic modifying compounds [111, 115, 116].

Due to the growing influence of epigenetic regulation on cell fate, an increasing number of studies have explored this approach to modulate EV therapeutic efficacy [117, 118]. Recently, it was demonstrated that epigenetic activation of osteoblasts substantially promoted the osteogenic potential of secreted EVs (Fig. 2) [119]. The authors induced hyperacetylation in osteoblasts using the HDAC inhibitor Trichostatin A, which augmented the epigenetic function within the cells, accelerating mineralisation. EVs isolated from these epigenetically modified cells were found to be significantly enriched in several pro-osteogenic factors (i.e. microRNAs and transcriptional regulating proteins). Importantly, the epigenetically-modified cell-derived EVs significantly enhanced the recruitment and mineralisation of recipient human bMSCs through the delivery of pro-osteogenic factors. Thus, epigenetic reprogramming of the parental cells could produce EVs of a more mature osteogenic phenotype, due to the enrichment of multiple bioactive factors that work synergistically to promote bone formation. Collectively, these studies highlight the potential of exploiting biomimetic epigenetic mechanisms to facilitate the manufacture of pro-regenerative vesicles for bone regeneration.

#### Hypoxia

During the process of bone healing, hypoxia is a critical environmental stimulus regulating tissue regeneration. In vivo oxygen concentrations range from 2 to 8%, whilst in vitro, cells are exposed to concentrations of 21% [120]. Several studies have reported the importance of hypoxic conditions within the bone defect in promoting the osteogenic differentiation of precursor cells and stimulating repair [121, 122]. As such, researchers have investigated the influence of inducing hypoxia as a biochemical stimulus to enhance the pro-regenerative capacity of secreted EVs (Fig. 2).

For instance, Liu et al. investigated the impact of hypoxia on the therapeutic efficacy of MSC-EVs on bone fracture healing. MSCs-derived from human umbilical cord tissue were subjected to either normoxic (21% O_2_) or hypoxic conditions (1% O_2_) [123]. The findings showed that hypoxic conditions significantly enhanced the quantity of MSC-EVs (~ 1.5-fold) when compared to vesicles procured from cells in normoxic conditions. Hypoxia-derived EVs promoted the recruitment and angiogenesis of ECs both in vitro and in vivo. Microarray analysis identified miR-126 to be upregulated within hypoxia-derived EVs, which promotes HUVEC angiogenesis by activating the SPRED1/Ras/Erk pathway. Liang et al. chemically-induced hypoxia in MSCs via treatment with dimethyloxaloylglycine (DMOG) that stimulates HIF-1α expression [124]. In this work, EVs derived from DMOG treated MSCs stimulated the angiogenesis of HUVECs by activating the AKT/mTOR signalling pathway. Moreover, the DMOG-MSC-EVs substantially promoted healing of critical-sized calvarial defects in rats by stimulating neovascularisation.

### Biophysical stimulation

**Fig. 3:**
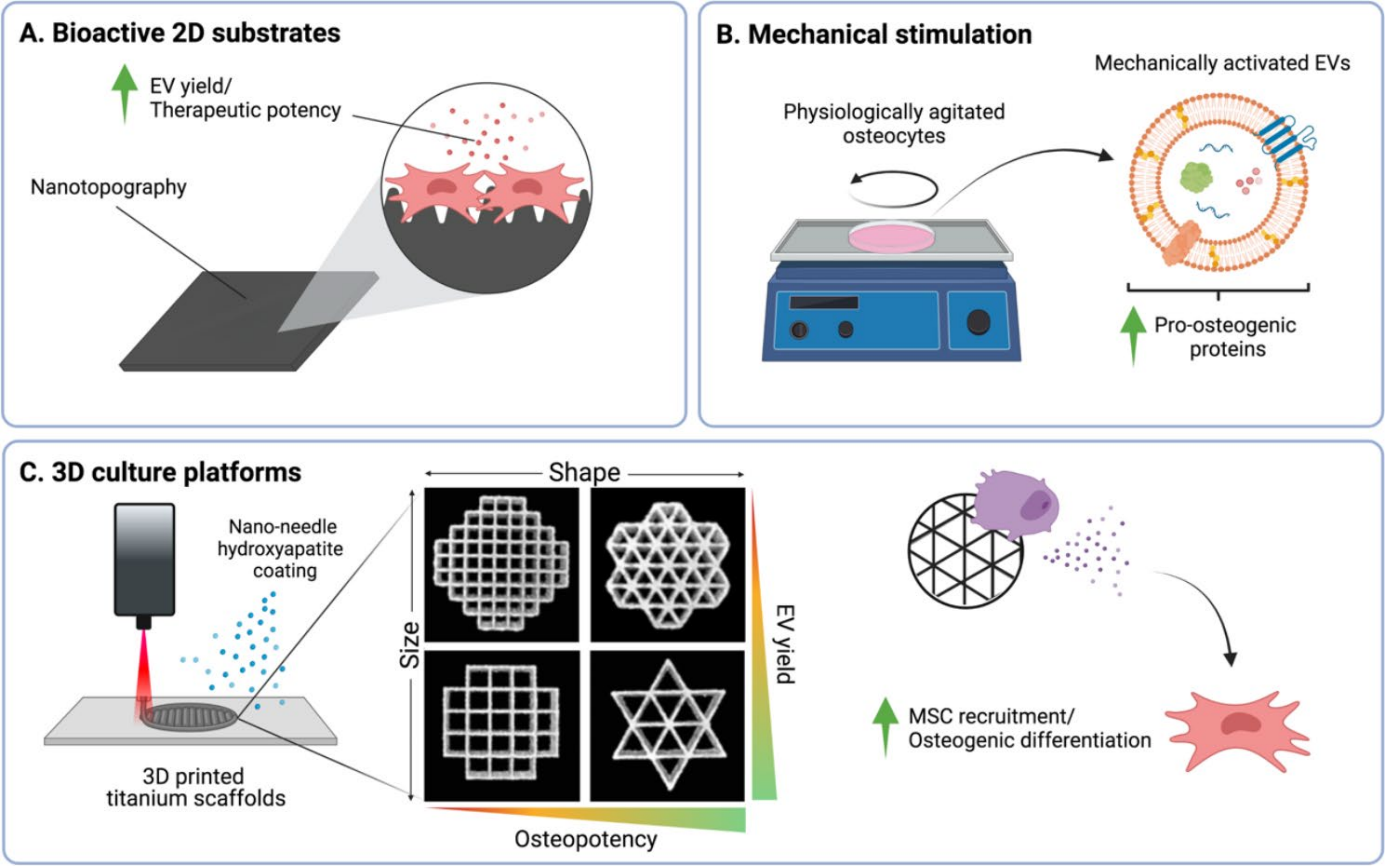
Biophysical stimulation to generate pro-regenerative EVs for bone repair. Strategies include (**A**) utilising bioactive 2D culture substrates, (**B**) mechanical stimulation and (**C**) biomimetic 3D culture platforms

To date, EVs are commonly procured from cells cultured on 2D planar tissue culture plastic, which does not replicate the complex physiological environment within the body [125, 126]. Hence, harnessing more physiologically relevant substrates could provide a platform to improve the production of pro-regenerative EVs targeted for bone repair. In addition to the cell’s spatial orientation within the bone microenvironment, they are subjected to biophysical stimuli such as topography, strain, pressure, and fluid shear, all of which have been shown to contribute to bone mechanoadaptation [127–129]. As such, biophysical stimulation has been exploited as an approach to modulate cell behaviour in bone tissue engineering applications [130]. In this section, we explore novel engineering strategies harnessing biophysical stimulation to enhance the production of pro-regenerative vesicles targeted for bone regeneration (Fig. 3).

#### Bioactive 2D substrates

Titanium alloys have been widely used in numerous orthopaedic applications due to their biocompatibility, mechanical properties and osteoinductivity [131, 132]. Due to these favourable properties, studies have investigated the use of these biomaterials as substrates for EV manufacture [133, 134]. For example, Ma et al. evaluated the impact of alkali and heat treatment to infer nanotopography on titanium surfaces and improve the osteogenic potency of MSC-derived EVs (Fig. 3) [133]. The findings showed that nanotopography (average roughness = 400 nm) accelerated bMSCs osteogenic differentiation when compared to polished titanium controls (average roughness = 40 nm). RNA sequencing demonstrated that the nanotopography increased the enrichment of pro-osteogenic microRNAs within secreted EVs as osteogenesis advanced. Moreover, when combined with a polydopamine coated 3D printed polyether ether ketone scaffold, the EVs-functionalised construct substantially promoted bone repair within a rabbit femoral condyle defect model. Zhang et al. adopted a similar approach, investigating the influence of small-scale topography, nano (50–200 nm) and micropits (10–50 μm) applied to titanium surfaces on augmenting the regenerative capacity of EVs derived from macrophages [135]. This small-scale topography titanium was found to stimulate polarisation to the M2 phenotype and the vesicles derived from these macrophages significantly promoted osteoblast mineralisation. The authors utilised RNA sequencing to identify enrichment of several microRNAs involved in regulating osteogenesis within the stimulated macrophage derived EVs. Similarly, Jin et al. reported the development of biomimetic hierarchical extrafibrillarly (EMC) or intrafibrillarly mineralised collagen (IMC), which differentially orchestrated macrophage polarisation to the M1 and M2 phenotype respectively [136]. Moreover, IMC substrates promoted the recruitment and osteogenic differentiation of MSCs to a greater degree when compared to EMC groups. Liu et al. isolated macrophage-derived EVs from these bioactive substrates and showed that IMC-EVs enhanced MSCs mineralisation when compared to EMC-EVs treatment [137].

#### 3D culture platforms

Within the regenerative medicine field, growing research has investigated harnessing 3D culture platforms to more closely replicate the cells native environment. Several studies have shown that cells in 3D culture exhibit enhanced osteogenic capacity when compared to cells on 2D surfaces [138–140]. Of the numerous classes of biomaterial systems available, hydrogels are widely used due to the ability to tune their physical and biological characteristics. Moreover, their capacity to replicate the cells native extracellular matrix is critical in promoting osteogenesis [141]. Recently, Yu et al. investigated the impact of culturing hPDLSCs within a collagen hydrogel on EV yield and osteoinductive potency [142]. The findings showed that there was a 2.5-fold increase in the number of EVs acquired from 3D culture when compared to 2D tissue culture. Moreover, vesicles derived from 3D culture significantly enhanced bMSCs proliferation, migration and osteogenic differentiation when compared to 2D-EVs and the EV-free group. Furthermore, these 3D-derived EVs combined with Matrigel accelerated bone healing within a rat alveolar bone critical defect model compared to the Matrigel alone group.

Although the use of hydrogel systems for the production of pro-osteogenic EVs have shown promise, the sourcing of naturally-derived biomaterials could suffer from batch and lot inconsistencies, which will hinder the reproducible manufacture of EVs from these 3D systems [143, 144]. Moreover, several studies have reported the capacity of EVs to bind to natural matrix components [145, 146], thus isolating vesicles from matrix-based materials may prove challenging. Hence, there is great precedence to generate 3D culture systems utilising materials and processes that can facilitate reproducible and scalable manufacture of pro-regenerative EVs targeted for bone repair. In a recent study, researchers investigated the use of 3D printed titanium scaffolds coated with nano-needle hydroxyapatite (nnHA) for the production of pro-osteogenic vesicles (Fig. 3) [147, 148]. Osteoblast seeded scaffolds exhibiting increased permeability (triangle pore shape, 1000 μm pore size), secreted an enhanced quantity of EVs (> 2-fold) when compared to the other scaffold designs (square pore shape, 500 μm pore size). Moreover, osteoblast-derived EVs from triangle pore scaffolds accelerated bMSCs mineralisation when compared to vesicles from other scaffold designs and 2D controls. Interestingly, nnHA scaffold coating further improved EV production when compared to uncoated scaffolds (> 3.5-fold), attributed to the ceramic promoting intracellular calcium signalling.

#### Mechanical stimulation

Mechanical stimulation has garnered increased attention as an approach to improve the osteogenic capacity of cells for bone augmentation strategies [149, 150]. Researchers have also attempted to replicate the physiological mechanical strain of cells in vivo, to enhance the production of EVs for regenerative medicine [151]. Eicholz et al. reported that osteocytes subjected to physiological fluid-flow resulted in the production of EVs that enhanced the recruitment and osteogenesis of bMSCs (Fig. 3) [78]. Moreover, proteomics analysis identified the upregulation of pro-osteogenic proteins such as Annexins within the mechanically-stimulated vesicles, which likely contributed to the EVs enhanced osteoinductive potential. Similarly, Morrell et al. showed that fluid-shear stimulated the production of pro-osteogenic EVs through the activation of calcium-dependent contractions within the cell [152].

Interestingly, studies have investigated the impact of mechanical stimulation on the secretion of pro-regenerative EVs when cultured in a 3D microenvironment. For instance, Yu et al. cultured hPDLSCs within a collagen/Fe_3_O_4_ nanoparticle composite hydrogel and subjected the construct to 20% magnetic-induced strain [153]. The authors observed that magnetic stimulation enhanced the biological potency of hPDLSCs-EVs, which promoted the recruitment and osteogenesis of bMSCs. Moreover, mechanically-stimulated vesicles accelerated the repair of alveolar bone defects in rats when compared to EVs derived from static cultures. Collectively, these studies highlight the considerable impact biophysical engineering has on the production of pro-regenerative EVs targeted for bone regeneration.

## Scalable manufacture of EVs

**Fig. 4:**
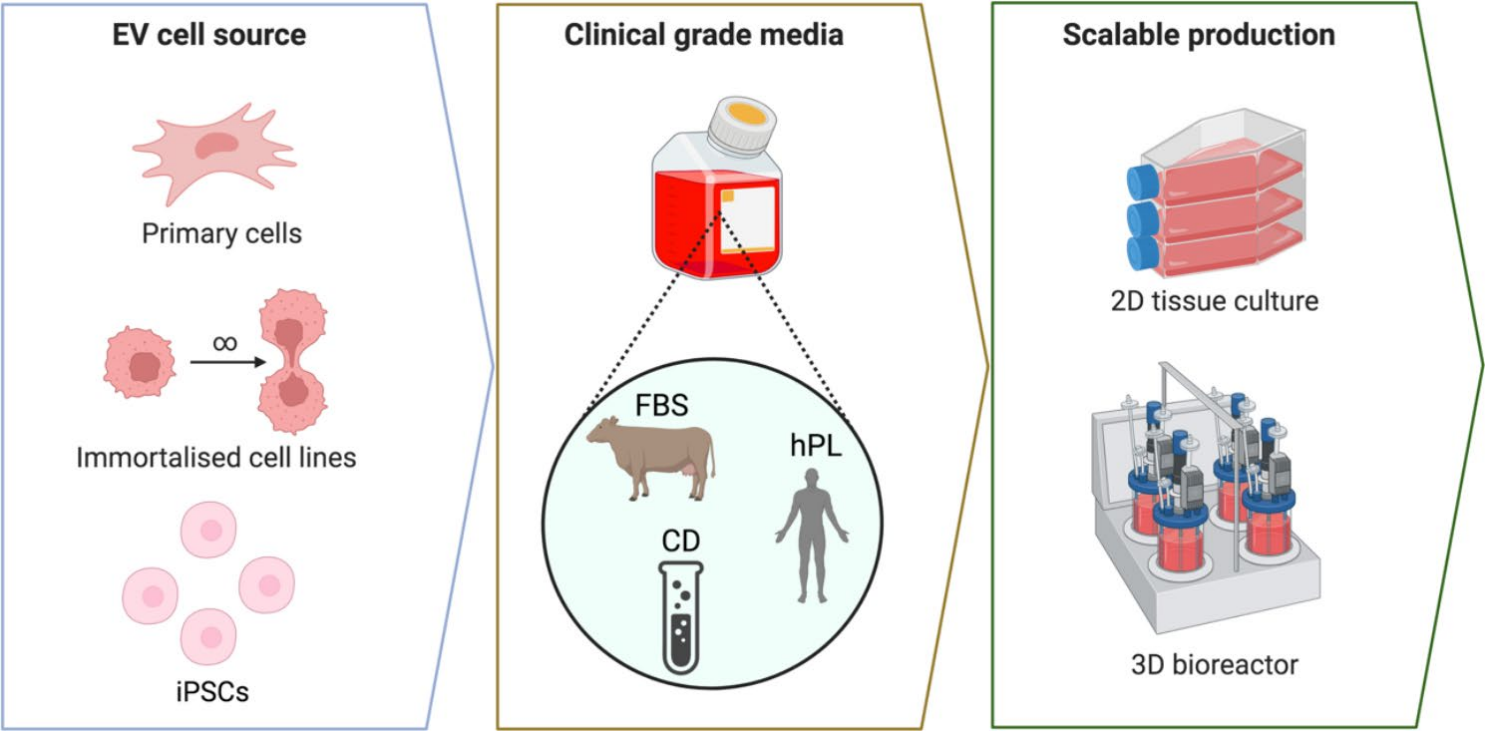
EV processing parameters in the scalable manufacture of pro-regenerative EVs for bone regeneration. Key components within the EV supply chain include using an appropriate EV cell source, the use of clinical grade media and harnessing compatible scalable systems. iPSCs - induced pluripotent stem cells, FBS - fetal bovine serum, hPL - human platelet lysate, CD - chemically-defined

Despite the promising osteoinductive capacity of EVs for bone repair, it is expected that large doses of vesicles are required to achieve regenerative effects clinically for both systemic and local EV administration [154]. This necessitates the development of robust manufacturing processes that could increase the consistency and scalability of EV production, which are currently lacking. To achieve clinically-relevant EV products, it is critical to employ the use of Good Manufacturing Practice (GMP)-compliant materials early in development to facilitate translation. EV-based products are a function of cell sourcing (donor, phenotype, expansion) and manufacturing parameters (culture conditions, production platform) [155]. Thus, it is critical to investigate the use of GMP-compatible materials and processes to facilitate clinical translation. This section will explore recent efforts to improve the clinical utility of major components in the EV supply chain, such as the use of an appropriate cell source, clinical grade media and scalable platforms (Fig. 4).

### EV cell source

There has been extensive research harnessing vesicles derived from primary cells. However, issues with cell sourcing, limited window of expansion, heterogeneity, donor and lot variations will hinder the production of therapeutically effective EVs at clinically relevant quantities [32, 155]. Thus, there is great incentive to manufacture EVs from cell lines that could enable a scalable and reproducible vesicle manufacturing solution as well as facilitating downstream processes (Fig. 4). This approach has already been utilised for the production of clinical grade stem cells for the treatment of central nervous system disorders [156]. It is important to determine whether cell lines are able to produce EVs of similar therapeutic efficacy compared to their primary cell counterparts during the scale up process. Chen et al. found that following the immortalisation of embryonic stem cell-derived MSCs via MYC overexpression, there was extensive modulation in cellular protein expression, which likely impacts secreted EV composition [157].

Another approach to generating EV producer cells is the use of induced pluripotent stem cells (iPSCs). These cells were first generated in 2006 by the transduction of four transcription factors, Oct3/4, Sox2, c-Myc, and Klf4 into adult fibroblast cells [158]. These iPSCs have the unlimited proliferative capacity of embryonic stem cells (ESCs), however, are not associated with the immunogenicity and ethical concerns of ESCs. Thus, iPSCs could provide an inexhaustible source of cells for the production of pro-regenerative EVs. Researchers have shown that these cells can produce up to 16 times more EVs when compared to vesicles derived from bMSCs, while exhibiting similar physiological functions [159]. Qi et al. reported that iPSC-MSC-EVs promoted the proliferation and osteogenic differentiation of MSCs derived from OVX rats in vitro [160]. Moreover, when combined with a β-TCP scaffold, these iPSC-MSC-EVs functionalised constructs enhanced bone regeneration and angiogenesis in an OVX rat critical-sized calvarial defect model in a dose-dependent manner when compared to the EV-free scaffold group.

### Clinical grade media

Another key factor affecting the production of therapeutically-relevant EVs is the use of clinical-grade medium (Fig. 4). Currently, the majority of EV studies utilised media supplemented with sera such as fetal bovine serum (FBS), which usually contains a large number of vesicles, therefore, ultracentrifugation is often conducted to remove EVs from FBS before onward use [161]. Although an effective strategy to remove contaminating EVs, it would be difficult to implement at a commercial scale as ultracentrifugation is more suited to scale out than scale up, which is cost intensive [162]. For the manufacture of cell therapies, researchers have started to shift towards the use of human platelet lysate (hPL) since it avoids the xenogeneic implications and supply issues of FBS [163]. Although its potential has been demonstrated, hPL is still derived from serum, contains EVs and may suffer from batch variability [155]. Thus, there is an urgent need for researchers to shift towards the use of chemically-defined (CD), xeno and blood-free culture medium that complies with the regulatory framework. Employing the use of compatible CD media will improve the reproducibility of EV production at scale. Several studies have reported the promise of CD medium for the generation of therapeutically effective EVs [164]. It is important to validate whether the CD medium is able to not only support cell expansion, but also allow for the production of EVs that exhibit similar therapeutic efficacy compared to the use of conventional non-CD media. Scheiber et al. investigated the impact of CD culture medium on the production of bMSCs-derived EVs [165]. When compared to conventional medium supplemented with FBS, bMSCs exhibited a substantially reduced doubling time when cultured in CD media. Moreover, bMSCs cultured in CD media exhibited 13-fold increase in EV quantity when compared to non-CD cultures. Interestingly, the authors found that RNA content within the CD-derived EVs were significantly reduced, with microRNA profiles shifting towards cell growth and proliferation within these vesicles. Similarly, Figueroa-Valdes et al. evaluated the use of regulatory complying medium, Oxium™ EXO, on the production yield and potency of MSC EVs [164]. Their findings revealed a ~ 4-fold increase in EV yield when umbilical cord-menstrual blood-derived MSCs were cultured in the CD-medium compared to non-CD FBS-free medium after 6 days of culture. Moreover, EVs derived from CD-cultures exhibited similar expression of vesicular markers, in vitro internalisation and in vivo systemic biodistribution compared to EVs from control medium.

### Scalable production

The acquisition of pro-regenerative EVs at large scales has the potential to transform the treatment of debilitating bone-associated disorders such as OP. The systemic administration of vesicles for OP has shown promise [48, 166], however, low yield of EVs from current manufacturing methods and variable dosing regimens has hindered their translation. In addition to systemic administration, the mass manufacturing of pro-regenerative EVs will significantly benefit the local administration of these vesicles for the repair of critical-sized bone defects/fractures. Thus, it is clear there is an urgent need to develop approaches to effectively scale the manufacture of pro-regenerative vesicles for bone regeneration.

Bioreactor platforms have been commonly utilised for the large-scale production of clinical-grade cells for therapeutic applications [167, 168]. As EVs are products of cells, recently researchers have investigated the use of these systems to facilitate the scalable manufacture of EVs (Fig. 4). Bioreactor systems allow for better control of environmental parameters such as oxygen, carbon dioxide, temperature, pH levels and nutrient/waste transport that together contributes to enhanced reproducibility and physiological recapitulation when compared to 2D flask culture [169]. Several studies have described the use of different bioreactor systems for EV scale up. For example, de Almeida Fuzeta et al. reported that MSCs cultured within a Vertical-Wheel™ bioreactor exhibited a 5.7-fold increase in EV quantity when compared to MSCs cultured on tissue culture plastic [154]. Similarly, Yan and Wu showed a 7.5-fold increase in EV yield when MSCs were cultured within a hollow-fibre bioreactor compared to conventional 2D culture [170]. As with the use of an appropriate cell source and culture medium, it is important to determine whether harnessing bioreactor platforms will adversely affect the therapeutic efficacy of EVs when generated at scale. Gobin et al. investigated the impact of hollow-fibre bioreactor culture on modulating the biological functionality of MSCs and their EVs [171]. The findings showed that bioreactor-cultured MSCs retained their expression of MSC markers and trilineage mesoderm differentiation capacity. Moreover, EVs derived from these bioreactor-cultured MSCs exhibited immune-regulatory constituents, indicating the suitability of this system for the scalable manufacture of therapeutic EVs. Although, there is growing evidence demonstrating the clear advantages of harnessing bioreactor systems for the scalable manufacture of EVs, there is currently no consensus on the type of bioreactors to use in the field. Stirred and hollow-fibre bioreactor systems have been the most utilised to date [155, 172]. A key advantage of the stirred systems is the capability to introduce microcarriers, allowing for greater control of cell numbers during the manufacture process and providing an additional degree of biophysical/chemical stimulation to modify cell/EV phenotype. However, the use of microcarrier-based bioreactor systems often result in large quantities of condition media, which requires extensive downstream processing for EV isolation/purification. A key benefit of hollow-fibre bioreactors is they allow for the retention of the EV product within the culture compartment, resulting is a more concentrated condition media, facilitating downstream processing [173]. A limitation of using these perfusion-based systems, as with most scale out approaches, are the restraints regarding cell number, where additional systems in parallel will be required to scale EV manufacture [172]. A critical difference between the stirred and hollow-fibre bioreactor systems is that there is shear stress applied to the cells in the stirred systems, whilst the hollow-fibre bioreactors eliminates this biomechanical stress. The use of this physiological parameter will be highly dependent on the cell type utilised and the EV product.

Microcarriers have been extensively employed for the manufacture of clinical-grade cells in conjugation with bioreactors systems [174, 175]. Due to their extensive use in cell manufacture, increasing studies have harnessed microcarrier-based bioreactor systems for EV production, with promising results observed [154, 176]. Three classes of microcarriers are commonly used for cell manufacturing, which are non-porous, microporous and macroporous in structure. The increased surface area of macroporous microcarriers directly resulted in higher cell yields in large scale cultures [177, 178]. In addition to the microcarrier structure, their underlying material is a crucial factor influencing cell behaviour. Microcarriers derived from synthetic sources (i.e. polystyrene, plastic, glass) can be manufactured in a reproducible manner and showed good mechanical properties, however, they often lack biological cues to facilitate cell adhesion and control cell function [179, 180]. Microcarriers derived from natural sources (i.e. gelatine, cellulose, agarose) are highly biocompatible and facilitate cell adhesion, but may suffer from batch and lot variability, whilst hindering clinical translation if derived from animal products. With increasing utilisation of additive manufacturing in regenerative medicine, fabricated microcarriers with increase controlled on the physical and chemical properties provides tremendous opportunity to tailor EV products during the scale up process [147, 181]. Thus, both the physical, chemical and manufacturing parameters of these microcarriers will be critical considerations for the scalable production of vesicle-based products for bone repair. Due to the additional biophysical/chemical cues microcarrier-based bioreactors systems provides when compared to 2D flask culture, it is critical to validate the transition of bioengineering approaches developed in 2D to 3D systems, to ensure the EV-based products retain their therapeutic potency during scalable manufacture. Additionally, it is important to determine if there are any degradation products from the microcarriers as this will affect downstream purification of these vesicles [155].

Thus, it is critical to assess the impact of the entire EV supply chain on the scalable and reproducible production of clinically effective vesicles for bone augmentation strategies.

## Therapeutic administration of pro-regenerative EVs for bone repair

### Systemic delivery

**Fig. 5:**
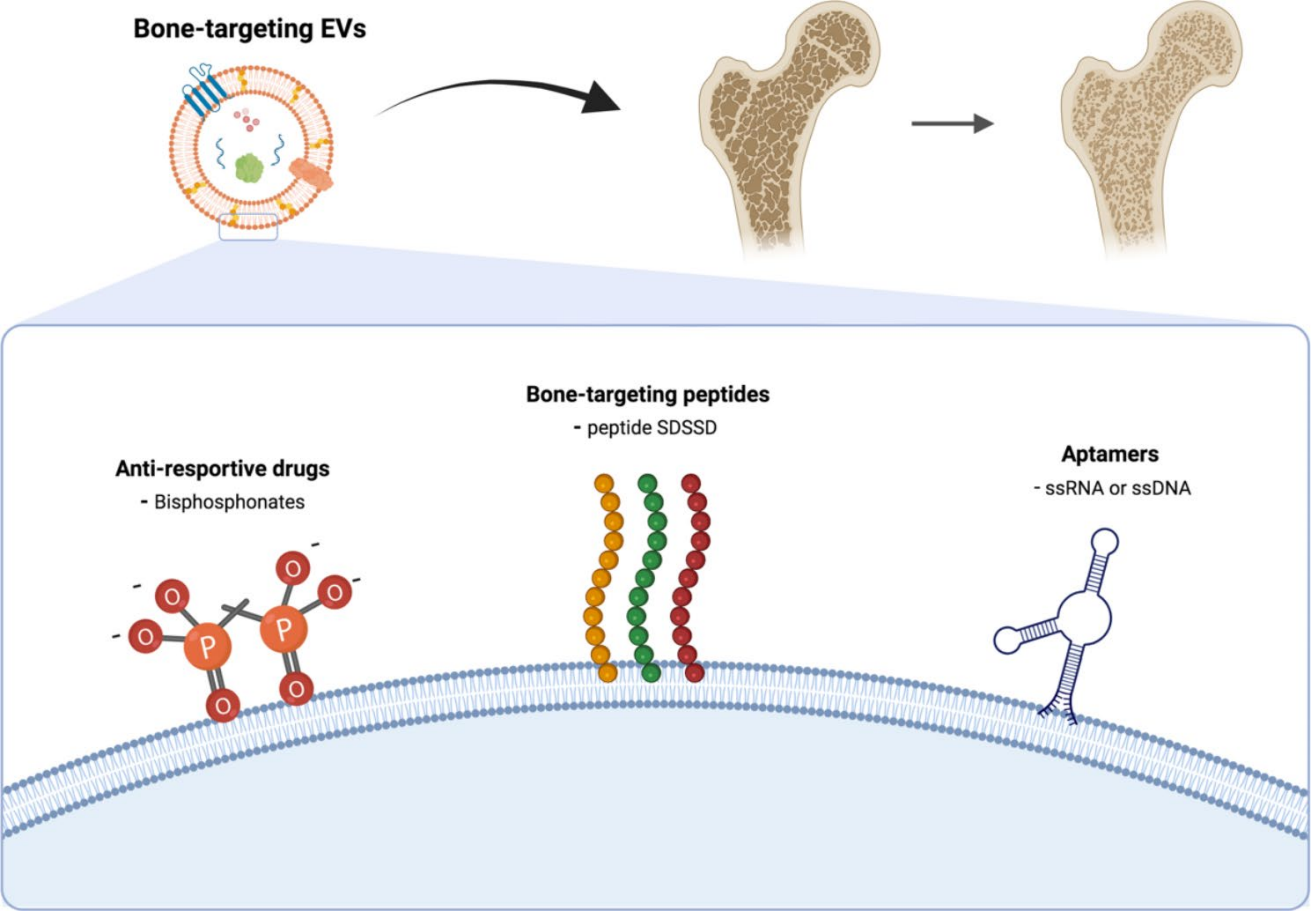
Schematic representation of surface modification strategies to promote the bone targeting efficacy of EVs for the treatment of systemic bone disorders. Surface modification approaches include the use of anti-resorptive drugs, conjugating bone-targeting peptides and functionalisation with aptamers

The demonstration of EVs pro-osteogenic capacity in vitro has led researchers to investigate their potential use for the treatment of systematic skeletal diseases such as OP. Although EV administration via intravenous injection for the treatment of OP has shown promise [160], the nanoparticles rapid clearance from systemic circulation hinders its therapeutic efficacy [182–184]. Moreover, natural unmodified EVs often exhibit insufficient tropism to the tissue of interest [185], hindering the desired therapeutic response of these nanoparticles. Thus, there have been extensive investigations to enhance the targeting capacity of systemically administered vesicles to bone tissues via surface modifications (Fig. 5).

#### Anti-resorptive drugs

One approach exploits the affinity of anti-resorptive drugs to bind to the mineral phase of bone tissue (Fig. 5). For instance, Wang et al. engineered a bone targeting EV by functionalising the bisphosphonate - alendronate on the surfaces of vesicles derived from murine MSCs [166]. In vitro these Ale-EVs exhibited high affinity to hydroxyapatite. When administered to OVX rats, Ale-EVs displayed enhanced accumulation in bone tissue when compared to the unmodified EVs. Moreover, Ale-EV treatment enhanced bone formation within OVX mice compared to groups treated with EVs and alendronate alone.

#### Bone-targeting peptides

In recent years, peptides have attracted increasing attention as targeting ligands due to their low immunogenicity, low cost and high specificity [186]. Cui et al. explored the use of bone-targeting peptides to improve the therapeutic efficacy of EVs secreted by MSC derived from IPSCs to treat OP (Fig. 5) [187]. The siRNA of Shn3 was loaded into EVs via electroporation, then conjugated with the peptide SDSSD (Ser, Asp, Ser, Ser, Asp) through a diacyllipid insertion method. This bone-targeting peptide binds to periostin, a protein expressed specifically on the surface of osteoblasts [188]. The engineered vesicle delivered the siRNA specifically to osteoblasts, inhibiting Shn3 expression and promoting osteogenic differentiation. Moreover, the modified EVs exhibited enhanced accumulation within mice bone tissue when compared to unmodified EV treatment. Importantly, the peptide-conjugated EVs significantly prevented OVX-induced bone loss in mice.

#### Aptamers

Several studies have investigated the use of aptamers, single-stranded RNA or DNA that are capable of folding into 3D structures and exhibit high affinity to specific targets [189, 190]. As such, aptamers are emerging as novel ligands to facilitate the targeting of pro-osteogenic EVs to appropriate tissues of interest (Fig. 5). For example, Luo et al. conjugated an MSC-specific aptamer to the surface of EVs derived from MSCs [191]. The authors showed that following intravenous injection in mice, the aptamer-conjugated EVs exhibited enhanced accumulation within the long bones when compared to unmodified EVs. Moreover, intravenous injection of aptamer-functionalised EVs enhanced bone mass in OVX mice and promoted bone healing in a femur fracture mouse model.

Although, there have been increasing investigations using vesicles for the repair of systematic skeletal diseases such as OP [166, 192], there is tremendous variation in the frequency of treatment utilised in the field [193]. Thus, it is critical to determine the efficacy of different EV treatment doses and frequency to provide increased pre-clinical evidence to support onward translation for specific bone injury/disease indication.

### Local delivery

For the repair of critical-sized bone defects, it is expected that a large quantity of EVs will be required to facilitate effective tissue regeneration. Due to issues with the administration of EVs via systemic routes and the manufacture of vesicles at large quantities, researchers have investigated locally delivering these vesicles directly into the bone defect in conjunction with an appropriate biomaterial [194–196]. This approach allows for the mechanical reinforcement of the bone defect, in addition to delivering a concentrated dose of pro-regenerative EVs to maximise their bioavailability and regenerative capacity. Moreover, locally delivering vesicles minimises potential off targets effects such as inducing ectopic bone formation.

**Fig. 6:**
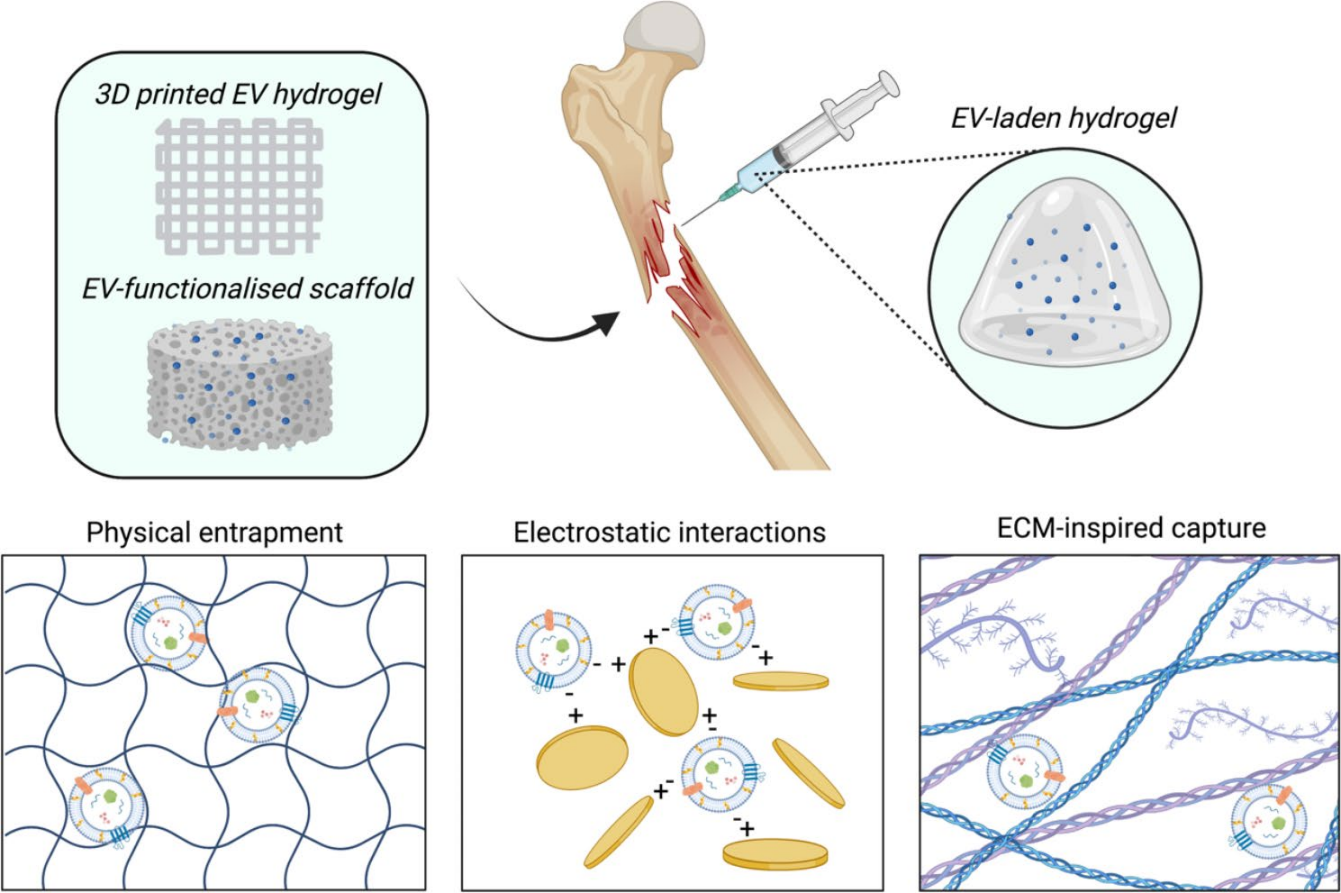
Overview of EV-biomaterial functionalisation strategies to improve the local retention and control delivery of pro-regenerative EVs targeted for bone fracture repair. EV functionalisation strategies include physical entrapment, electrostatic immobilisation and interaction with ECM-mimetic components

As EVs exhibit a plethora of different physiochemical properties [197, 198], this provides researchers with numerous different approaches to enhance the bioavailability of these nanoparticles through interactions with different biomaterial systems (Fig. 6).

#### Physical entrapment

The porosity of a biomaterial influences several factors such as cellular infiltration, proliferation, differentiation and nutrient/waste transport [199, 200]. Additionally, the biomaterials porosity and its associated degradation rate are important parameters impacting the release rate of encapsulated bioactive factors [201, 202]. Several studies have harnessed the capacity to physically immobilise EVs within biomaterial systems as a method to facilitate the delivery and sustained release of EVs (Fig. 6). Born et al. investigated the influence of photo-crosslinker concentration on the release kinetics of MSC-EVs from gelatin methacryloyl (GelMA) hydrogel [203]. The findings showed that using a higher crosslinker concentration resulted in a hydrogel with a smaller average pore size. Moreover, EV release kinetics from these constructs were significantly reduced when compared to more porous gels, likely due to the influence of macromer concentration on GelMA porosity and degradation kinetics.

Although this approach has shown promise, biomaterials with rapid degradation rates may have a detrimental impact on the stabilisation of the defect, a critical aspect for the repair of load-bearing tissues [204, 205]. Moreover, the biomaterial porosity and effects on construct biomechanics are known to be essential physiological cues to promote bone formation [206, 207]. Therefore, it is important to fully investigate the impact of physically immobilising EVs within biomaterials on key biomechanical processes involved in bone fracture healing.

#### Electrostatic interactions

Another approach to modulate the release of EVs from biomaterials is electrostatic interactions. Studies have reported that vesicles exhibit an overall negative surface charge, thus harnessing biomaterials that possess positively charged groups provides an approach for EV immobilisation [208]. For example, studies have harnessed the positively charged polymer chitosan as an approach to deliver EVs for different regenerative applications [145, 209].

The synthetic nanosilicate Laponite (LAP) has been reported to exhibit a broad affinity to bioactive molecules due to their positive rim charge and negative surface charge [210, 211]. Moreover, these nanoclay particles have been shown to promote bone formation due to the osteoinductive potency of their degradation products [211, 212]. Recently, researchers have investigated harnessing LAP to control the release of epigenetically-activated osteoblast-derived EVs from a GelMA hydrogel for bone repair (Fig. 6) [213]. The authors showed that LAP significantly enhanced the retention of osteoblast-derived EVs within the hydrogel in a dose-dependent fashion. The GelMA-LAP composite combined with the epigenetically-activated EVs was found to significantly promote the recruitment and mineralisation of human bMSCs.

#### ECM-inspired biomaterials

EVs have been reported to bind to native extracellular components found within the bone matrix, thus providing a biomimetic platform to facilitate the local delivery of pro-regenerative vesicles for bone regeneration (Fig. 6) [146, 214]. For example, Narayanan et al. demonstrated that EVs derived from MSCs were able to effectively bind to type I collagen and fibronectin, two key components of the bone extracellular matrix (ECM) [214]. Similarly, Nieuwoudt et al. described the functionalisation of electrospun polycaprolactone scaffolds with ECM proteins collagen and fibronectin to enhance mechanically stimulated osteocyte-derived EV binding [146]. The findings showed that both collagen type I and fibronectin scaffold coating significantly improved EV retention within the construct. Moreover, EV-functionalised collagen-coated scaffolds substantially improved human bMSCs mineralisation when compared to collagen-coated and uncoated constructs [215].

In a similar approach, researchers have developed an injectable ECM-mimetic hydrogel utilising chitosan and collagen to facilitate EV controlled release for bone repair [145]. Chitosan was selected due to its polysaccharide unit structurally resembling glycosaminoglycans, a key component of the bone matrix [216]. Moreover, due to chitosan’s positive charge [217], this allows for the electrostatic interaction with the negative surface of EVs. Collagen has been reported to bind to EVs via integrins and annexin proteins found on the nanoparticles surface [218, 219]. The findings showed collagen type I and chitosan incorporation differentially impacted cellular proliferation and osteogenic differentiation of encapsulated cells. Importantly, EV release kinetics from the composite hydrogel could be tailored by altering the proportion of collagen and chitosan. EVs released from the ECM-mimetic hydrogel enhanced the recruitment and mineralisation of bMSCs.

## Future perspectives and challenges

**Fig. 7:**
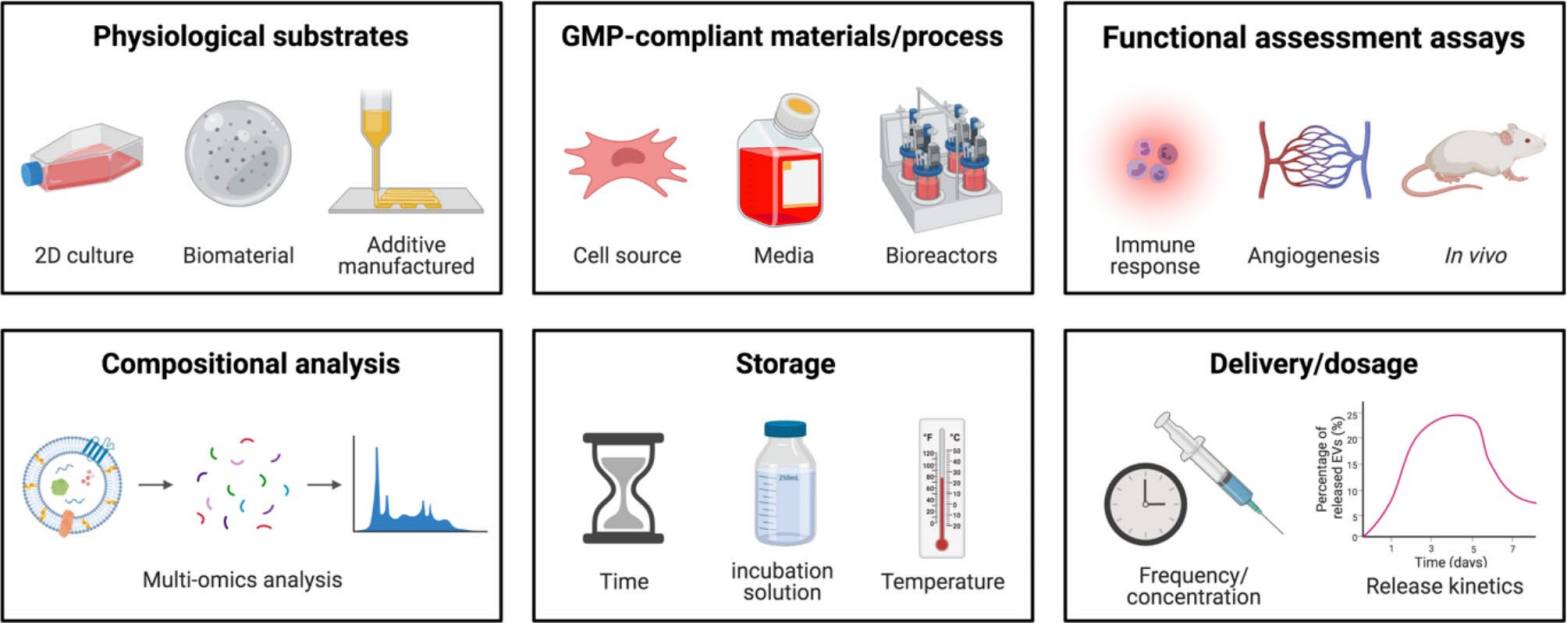
Key challenges in the EV supply chain hindering the translation of clinically-viable EV-based products targeted for bone regeneration

There is a growing body of evidence highlighting EVs as a promising acellular but biological approach for the treatment of bone disorders. Although these nanoparticles have shown their potential, from reviewing the current state of the literature, it is clear there are several challenges hindering the clinical translation of vesicles for bone repair (Fig. 7).

To facilitate the translation of pro-regenerative vesicles to the clinic, it is essential to elucidate EV composition to determine the “active drug substance(s)” involved in mediating the vesicles’ biological activity. Although increasing efforts in recent years have employed multi-omic approaches to identify key bioactive factors (i.e. lipids, proteins, metabolites, nucleic acids etc.), the majority of research has procured EVs from cells cultured on 2D tissue culture plastic, a highly artificial environment [125, 220]. Researchers have shown that EVs derived from cancer cells cultured in a 3D environment exhibited a 96% RNA similarity to natively-derived EVs, compared to 80% similarity to EVs from 2D culture [221]. Thus, harnessing biomimetic in vitro systems would result in the procurement of EVs that exhibit an in vivo-like composition, allow for a more accurate identification of their “active drug substance(s)” and mechanisms of action. This will also facilitate the development of critical quality assurance markers to ensure lot-to-lot consistency of manufactured EVs.

As EVs have been shown to consist of multiple different biological constituents, they may be able to effect bone regeneration through several pathways, overcoming one of the limitations of current clinically available growth factors [222]. Furthermore, due to the synergistic role of EVs in modulating processes such as cell recruitment, immunomodulation, angiogenesis and osteogenesis in bone healing [223], it is critical to elucidate the biological function of pro-regenerative EVs in each of these critical physiological pathways. Thus, due to the multi-functional role of EVs on bone remodelling, it is recommended that combinatorial potency assays should be employed.

To date, there is no consensus on a standardised approach to manufacture pro-regenerative EVs at scale. As it is expected that a large quantity of EVs will be required for the treatment of bone disorders, the use of bioreactor systems for EV manufacture is a logical step toward translation. As the scalable manufacture employing bioreactor systems is cost intensive, it is critical to use GMP-compatible materials and processes early in research and development to facilitate the translation of EV-based products. Moreover, it is key to confirm whether the transition to scalable manufacture adversely impacts the therapeutic potency of EVs when produced at large quantities. This transition to large scale production will ultimately be beneficial to the sustainable manufacture of these pro-regenerative vesicles. An in-depth overview of critical challenges facing the broader EV field for the large scale production of EVs can be found in the “massivEVs” ISEV report [224]. In addition to the scalable manufacture of EVs targeted for bone repair, another critical challenge is the influence of different isolation and purification procedures on the clinical efficacy of the bioengineered EV-based therapies. Thus, it is critical to determine the influence of different isolation and purification approaches and the potential impurities or co-isolates on the therapeutic efficacy of a bioengineered EV therapy.

As highlighted in our review, there has been extensive research harnessing novel bioengineering strategies to promote the therapeutic efficacy of EVs for the treatment of bone disorders. Although their promise has been demonstrated, it is critical to ensure the robustness, reproducibility and scalability of these approaches in order to facilitate the translation of these smart nanoscale therapeutics to the clinic.

In addition to the scalable manufacture of these nanoparticles, there is currently a lack of standardised quality assured functional assays to ensure appropriate batch and lot consistency. It is important to develop GMP appropriate functional in vitro assays that offer enhanced reproducibility, high-quality quantification and avoids confounding biological processes. The Minimal Information for Studies of Extracellular Vesicles from The International Society for Extracellular Vesicles provides guidelines for EV manufacture [225], however, these are often too generalised for the production of EVs targeted at a specific clinical application. Moreover, it is crucial to assess the safety and efficacy of manufactured pro-regenerative EVs in vivo within appropriate models of bone disease. Depending on the vesicle’s specific clinical inclination, it is important to evaluate the effects of different dosages and administration frequency to provide increased pre-clinical evidence to support onward translation. Additionally, it is expected that EV products will exhibit differential regenerative capacity in models of impaired healing (i.e. osteoporotic/aged models) when compared to defects in healthy subjects, thus, it is crucial to effectively define the target clinical use for a specific EV based product.

There has been increasing research investigating the local delivery of EVs within biomaterials systems, to enhance their bioavailability and maximise their therapeutic response. Although promising results have been observed, it is crucial to determine the impact of the biomaterial on facilitating EV-induced bone formation following administration. Moreover, optimisation of the vesicles dosing regimen in vivo is critical to maximise the nanoparticles therapeutic response, in addition to informing appropriate manufacturing batch requirements for certain clinical inclinations.

Finally, the standardisation of storage methods is a critical step towards the translation of EVs [226]. Thus, it is important to systematically investigate the influence of different storage parameters (i.e. temperature, storage solution, pH, time etc.) on the therapeutic potency of EVs clinically.

## Conclusion

While great developments have been made in elucidating the role of EVs in regulating bone remodelling, there are still several unmet challenges hindering the translation of clinically-viable vesicles for bone regeneration. This has propelled intensive investigations into novel bioengineering approaches to improve EVs’ therapeutic efficacy for bone repair. In this review, we highlight strategies harnessing biochemical/biophysical stimulation, GMP-compatible scalable production and maximising in vivo efficacy. We trust that continued progress in these areas will streamline the transition of promising bench-side results to clinically-viable smart nanoscale therapies for the effective treatment of bone disorders.

## Data Availability

Not applicable.
